# Getting the job done right requires the right tools: Increased CAR T cell efficiency with improved packaging cell line

**DOI:** 10.1016/j.omton.2024.200901

**Published:** 2024-11-15

**Authors:** Sarah Mathieu, Nicolas A. Gillet

**Affiliations:** 1 Namur Research Institute for Life Sciences (NARILIS), Department of Veterinary Medicine, University of Namur, Namur, Belgium

## Main text

Chimeric antigen receptor (CAR) T cells are preferentially engineered using γ-retroviruses and lentiviruses, two members of the *Retroviridae* family. These retroviral-based vectors are commonly produced by cotransfection of HEK293T cells with three plasmids encoding, respectively, the Pol and Gag proteins, vesicular stomatitis virus (VSV)-glycoprotein, and CAR transgene. In this issue of Mo*lecular Therapy Oncology*, Swanson et al. highlighted the presence of APOBEC3 (A3)-mediated mutations in the CAR transgene in CAR T cells. These mutations are believed to be responsible for the reduced efficacy of CAR T cells observed following lentivirus production in A3-expressing HEK293T cells. The authors suggest exploring the cellular mutator pathways activated during retrovirus production and creating producer cell lines that are knockouts (KOs) for these cellular mutators.^1^

A3 proteins are a family composed of seven antiviral enzymes (A3A–A3H). These proteins convert cytosine to uracil in single-stranded DNA and RNA of viral genomes. An A3 evolutionary footprint was discovered in a wide range of viruses, including reverse-transcribing viruses such as HIV-1, HIV-2, HTLV-1, and HBV.^2^ A3D, A3F, A3G, and A3H proteins have been demonstrated to restrict HIV-1 in the absence of the Vif accessory protein, as reviewed in Feng et al.^3^ This inhibition is associated with their incorporation into the viral particles. The effect of A3B on HIV-1 remains a subject of debate. Indeed, Bandarra et al. demonstrated that A3B is packaged into the viral particles of HIV-1 but appears to have little impact on its replication.^4^ In contrast, Doehle et al. provided evidence that A3B significantly inhibits HIV-1 infectivity and is responsible for the introduction of numerous G-to-A mutations in the proviral genome.^5^ Importantly, the A3 proteins are also able to target exogenous foreign DNA like transfected plasmids and drive their degradation.^6^

The study conducted by Swanson et al. primarily aimed to evaluate whether the lentivirus production system could induce the expression of A3s. The transfection of HEK293T cells with the three plasmids used to make lentiviruses demonstrated that while transcripts for A3B, A3C, and A3G were upregulated, only A3B showed an increase at the protein level. A3F transcription was also upregulated, but the authors did not report A3F protein levels, probably due to the fact that a good commercial antibody against A3F is still not available. It might be interesting to evaluate whether A3F is also upregulated at the protein level, as A3F is known to restrict HIV-1 and thus might also impact lentiviral vector production. Several mechanisms could be responsible for the upregulation of A3B protein in packaging cells. The mere presence of plasmid DNA in the cytoplasm and nucleus of packaging cells could trigger A3B expression. In this case, avoiding A3B expression would require the use of A3B-KO packaging cells. Alternatively, the increase in A3B protein level could be the result of the production of some viral proteins (the authors showed that A3B protein levels increase for several days after transfection). In this event, one could avoid A3B expression by modifying the viral vector without the need to change the packaging cell line.

The sequencing of the single-chain variable fragment (scFv) of the CD19 CAR gene present in the CAR plasmid, in the resulting lentiviral particles, and as inserted proviruses in the CAR T cells revealed the presence of characteristic G-to-A mutations. As no A3 proteins were detected in the receiving T cells, the authors proposed that the mutations observed in the final CAR T cells came from mutagenic activities in the producer HEK293T cells and during vector retrotranscription. Purification of CAR lentiviral particles on a sucrose gradient demonstrated the packaging of A3B into viral particles. Assessing the ability of A3B to be packaged into viral particles is highly significant. Indeed, A3s have been shown to deaminate the viral minus-strand cDNA during reverse transcription in the newly infected cells. Again, as the upregulation of A3F transcripts has been observed, it would be interesting to test whether A3F could be packaged into viral particles. Indeed, the A3 mutations in the scFV were found in the sequence motif 5′-TCT-3′ which is the sequence motif targeted by all A3 proteins except A3G. Some mutations could therefore be attributed to A3F.

Finally, Swanson et al. investigated whether the upregulation of A3B protein could impact the lentivirus titer and reduce the efficacy of CAR T cells in killing cancer cells. For this, they compared the lentiviral titer obtained from parental HEK293T cells with those from A3B-overexpressing HEK293T cells. They observed a lower viral titer when lentiviruses were produced in the A3B-overexpressing cells. The authors proposed that the reduced viral titer is the consequence of the A3B mutagenic activity. This is certainly the most likely explanation, as deamination of the plasmid DNA and/or proviral DNA can lead to their degradation. It would be interesting to test whether an enzymatically inactive A3B can still be responsible for the reduced lentiviral titer. Of note, a study demonstrated that A3B inhibits certain viral promoters such as the cytomegalovirus (CMV) and SV40 promoters.^7^ Since Gag and Pol protein expression relies on the CMV promoter, the reduced lentiviral titer could also be attributed to a decreased production of viral proteins, leading to subsequent lower lentiviral particle production.

The efficacy of CAR T cells was evaluated by exposing the cells to tumor cells. The experiment revealed a decreased production of interferon (IFN)-γ and a reduced ability to kill tumor cells when CAR T cells were engineered with lentiviruses produced in A3B-overexpressing cells. The reduced efficacy could be attributed to an increased proportion of defective lentiviruses or the presence of mutations in the CAR gene, as well as changes in the proviruses’ insertion sites. Indeed, Ajoge et al. demonstrated that A3s can introduce mutations into the long terminal repeats of lentiviruses and bias their insertion into less transcriptionally active regions of the genome.^8^ Crucially, the authors showed that lentiviruses produced in the A3B-KO cell line contained less mutations than those produced in the parental cell line. Moreover, the production of IFN-γ was higher and the amount of remaining tumor cells was reduced when tumor cells were exposed to CAR T cells engineered with lentiviruses produced in A3B-KO HEK293T cells. This last experiment demonstrate that A3B-KO packaging cells show superior ability to produce productive lentiviral particles.

The study performed by Swanson et al. highlights the importance of investigating the antiviral mechanisms activated following clinical-grade virus production, particularly the mutagenic antiviral mechanisms. Indeed, in addition to the engineering of CAR T cells, viruses are also used as backbones for gene therapy or vaccines or as oncolytic viruses. The findings from Swanson et al. will be highly valuable in advancing CAR T cell engineering. It would be interesting to test whether γ-retroviruses used in CAR T cell production also elicit an A3 response and whether a total A3 KO could further enhance lentivirus and γ-retrovirus production, subsequently improving CAR T cell efficacy.

Lastly, HEK293T cells are used to produce not only lentiviral vectors but also adenoviral and AAV vectors. We recently demonstrated that A3B is an adenoviral restriction factor, introducing mutations in the viral progeny.^9^ Thus, HEK293T A3B KO might also prove useful for adenoviral vector production.

## Acknowledgments

S.M. is PhD fellow supported by F.R.S-FNRS FRIA grant n°4000865.

## Declaration of interests

N.A.G. is a co-holder of a patent relative to adenoviral vector production in APOBEC-deficient cells: GILLET Nicolas, LEJEUNE Noémie. (2022). APOBEC-DEFICIENT CELLS FOR ADENOVIRAL VECTOR PRODUCTION. PCT Application N°WO2024/126795A1, World Intellectual Property Organization.
